# Urticaria and mimickers of urticaria

**DOI:** 10.3389/falgy.2023.1274031

**Published:** 2023-09-28

**Authors:** Jie Shen Fok, Constance H. Katelaris

**Affiliations:** ^1^ Department of Respiratory Medicine and General Medicine, Box Hill Hospital, Eastern Health, Melbourne, VIC, Australia; ^2^ Monash Lung, Sleep and Allergy/Immunology, Monash Medical Centre, Melbourne, VIC, Australia; ^3^ Eastern Health Clinical School, Monash University, Melbourne, VIC, Australia; ^4^ Department of Medicine, Immunology and Allergy Unit, Campbelltown Hospital, Sydney, NSW, Australia; ^5^ School of Medicine, Western Sydney University, Sydney, NSW, Australia

**Keywords:** urticaria, mimickers of urticaria, cutaneous mastocytosis, erythema multiforme, urticaria vasculitis, erythema marginatum, urticarial dermatitis, bullous pemphigoid

## Abstract

Urticaria is a common skin condition encountered across various specialties in medicine, especially in dermatology and allergy/immunology practice. It has a heterogeneous presentation hence it is unsurprising that many skin conditions may be confused with urticaria. Urticaria may present as acute or chronic urticaria, the latter can be further categorised into chronic spontaneous and chronic inducible. In this article, we explore, explain, and summarise various skin lesions that are considered mimickers of urticaria, to promote understanding of each of the conditions highlighted, improve recognition, and reduce misdiagnosis.

## Introduction

**Figure 1:**
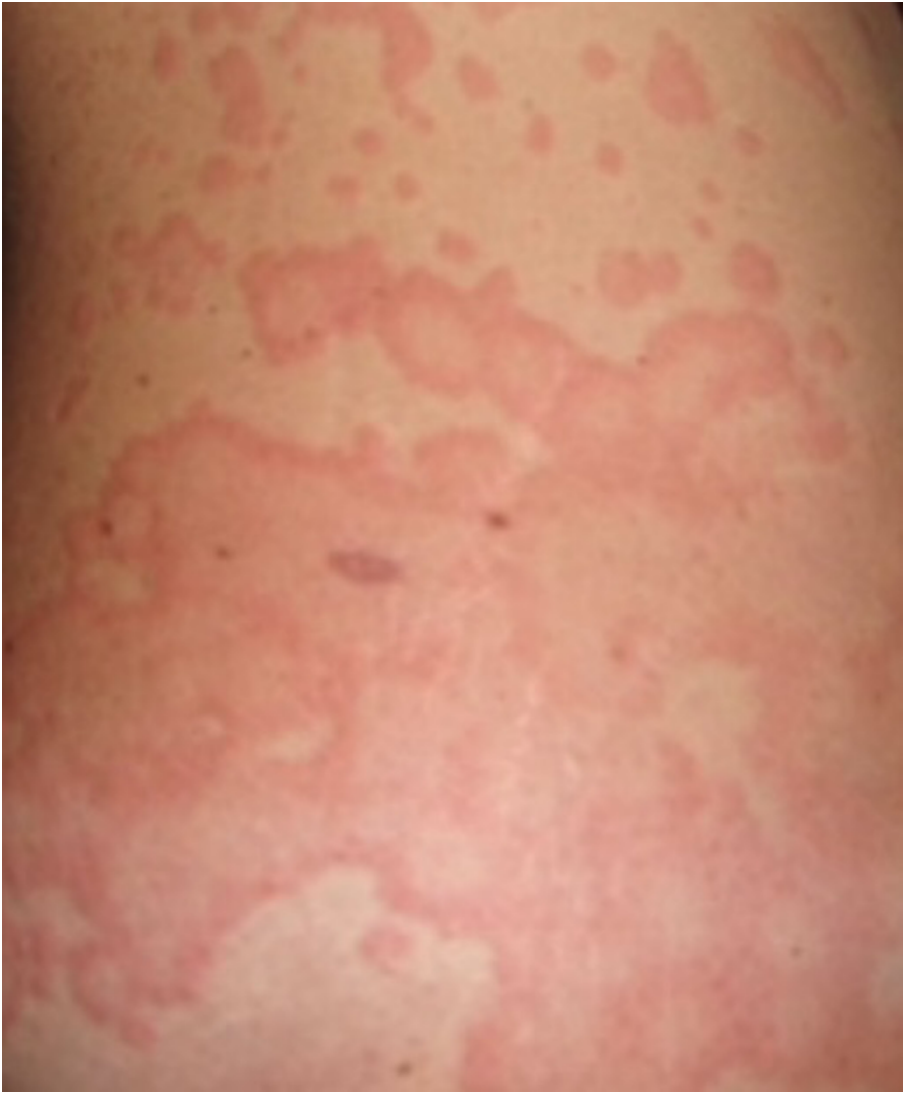
An urticarial wheal.

Urticaria is a common presentation in general practice and a common referral to clinicians including dermatologists, allergists, and immunologists. It is an inflammatory skin disorder characterised by wheals, hives, and often intractable itch, with an estimated lifetime prevalence of 20% globally (1). A typical urticarial wheal has a circumscribed superficial central swelling that may present with various sizes and shapes, surrounded by erythema, often with rapid appearance, and may appear on different locations of the body, including the face, trunk, and limbs (Figure 1). It has a fleeting nature, with resolution to its normal appearance within 24 h. The intensity of the itch is known to fluctuate during the course of its appearance. Angioedema, a swelling with similar pathogenesis, involves the submucosal, lower dermis, and subcutaneous tissues. It may occur suddenly, with pronounced erythematous or skin-coloured swelling (2). Resolution of angioedema is slower than urticarial wheal and may take up to days (e.g., 48–72 h). Urticaria and angioedema may occur alone or together, in both paediatric and adult populations.

Atypical urticarial lesions may appear more indurated, more discoloured, dusky, or bruised in appearance, and last longer. The lesions may heal with an eventual hyperpigmented mark. Instead of pruritic, they are painful. Angioedema is typically absent. Systemic features may coexist, including arthralgia, fever, and malaise.

Urticaria can be categorised as acute or chronic. Acute urticaria is recurrent flares of hives lasting less than 6 weeks. It occurs most often in children, in the setting of upper respiratory viral infection in most cases (3). In other settings, acute urticaria presents as part of the spectrum of an acute allergic reaction, caused by drug allergy, food allergy, or Hymenoptera sting allergy. Commonly implicated drug causes in real-life practice include penicillin and non-steroidal anti-inflammatory drugs. Acute urticaria and angioedema are also manifestations of anaphylaxis, with progression to respiratory, cardiovascular, or gastrointestinal involvement following exposure to an allergen that triggers a generalised immune response with the release of various chemical mediators.

Chronic urticaria is defined as the appearance of hives occurring on most days of the week lasting for more than 6 weeks. A majority of chronic urticaria cases are chronic spontaneous urticaria (CSU), with two distinctive subtypes being recognised: autoallergic CSU (type 1 autoimmunity) with IgE auto-antibody involvement and autoimmune CSU (type IIb autoimmunity) with IgG auto-antibody involvement (4, 5). Chronic inducible urticaria (CIndU), on the other hand, is less prevalent than CSU and accounts for approximately 13% of chronic urticaria cases. It is also known as physical urticaria, aptly described because of a physical trigger that induces the appearance of wheals. The most prevalent CIndU types are symptomatic dermographism, cold urticaria, and cholinergic urticaria.

There is, of course, a list of differential diagnoses to be considered for CSU. These include CIndU, Schnitzler syndrome, cryopyrin-associated periodic syndrome, Still's disease, and urticarial vasculitis, with specific questions and aspects of physical examination that could guide further evaluation before a final diagnosis is determined (6).

There are also several skin lesions with morphology that resemble an urticarial lesion, which may pose challenges to even the most experienced clinician. Here, we discuss a list of cutaneous lesions that may be regarded as mimickers of urticaria to improve understanding of a common presenting problem in dermatology or allergy/immunology practice.

## Urticaria pigmentosa

Mastocytosis is a clonal disorder characterised by abnormal mast cells in various organs, including the skin, bone marrow, liver, and gastrointestinal tract. It is the result of abnormal expansion and focal accumulation of neoplastic mast cells driven by a gain-of-function mutation in KIT, a membrane protein that keeps growth and activation of mast cells in check. The most common mutation described is KIT D816V, though there are other less commonly described mutations. The spectrum of mastocytosis presentation is heterogeneous, ranging from cutaneous to systemic involvement. Cutaneous mastocytosis (CM) is largely benign, whereas systemic mastocytosis often has serious implications, particularly increased risks for anaphylaxis and lymphoproliferation.

CM is further categorised into maculopapular cutaneous mastocytosis (also known as urticaria pigmentosa), diffuse cutaneous mastocytosis, and mastocytoma of the skin. Most cases of CM occur in childhood, and in most cases, lesions usually regress by puberty (7). In most cases, bone marrow examination is not routinely required unless there are features that suggest systemic mastocytosis or lymphoproliferative neoplasm (8).

Urticaria pigmentosa was first reported in 1,869 in a 2-year-old child who presented with urticaria with a brownish stain (9). It was not until 1,878 that the term urticaria pigmentosa was coined (10). It is by far the most common form of CM, characterised by macules and papules, hence the aptly named maculopapular CM. It may have a brownish discolouration, with two distinctive variants recognised so far. The monomorphic variant is seen in both children and adults. The polymorphic variant is usually observed in childhood and has a tendency to disappear by puberty. A key feature of UP is a positive Darier's sign, which is considered a pathognomonic sign for CM, named after the 19th-century French dermatologist Ferdinand-Jean Darier. Darier's sign is positive when a CM lesion is gently rubbed or stroked, resulting in local itch and whealing within a few minutes. It is postulated that the phenomenon is caused by the degranulation of dermal mast cells with the release of mediators.

Skin biopsy is characterised by increased mast cell numbers in the papillary dermis and around blood vessels. Immunochemistry staining with mast cell tryptase antibody helps detect mast cell infiltrates. Using CD117 immunostaining, the mast cell distribution pattern and the percentage of mast cells in the inflammatory infiltrates can be determined (11). The role of skin biopsy is also important in prognosticating UP. The monomorphic variant is associated with sparing of the papillary dermis from mast cell infiltration. On the contrary, mast cell density in the papillary dermis has been found highest in the polymorphic variant and diffuse CM (12).

## Erythema multiforme

Erythema multiforme (EM) is a cutaneous and mucosal hypersensitivity reaction triggered by a stimulus. Infectious aetiology accounts for most cases of EM, with herpes simplex virus (HSV) being the most common cause in adults (13). Mycoplasma and Epstein-Barr virus are other commonly implicating organisms. Drug causes account for the remaining cases in clinical practice, including beta-lactam antibiotics, sulphonamide antibiotics, and anti-convulsants such as phenobarbital.

The emergence of the novel Coronavirus disease-19 (COVID-19) infection has seen cases of EM being reported as a cutaneous complication, either to the infection itself or to the vaccine (14, 15). EM due to COVID-19 vaccination occurred most often after the first dose, most commonly with mRNA vaccines in one study (15). In another study, EM was more commonly reported in COVID-19 infection compared to COVID-19 immunisation (16). Small numbers of cases make it challenging to establish a causal relationship. Numerous other vaccinations including combined mump, measles, and rubella vaccine, combined diphtheria, tetanus, and pertussis vaccine, varicella vaccine, pneumococcal vaccine, smallpox vaccine, and influenza vaccine have all been reported to cause EM in the Vaccine Adverse Event Reporting System (17).

**Figure 2:**
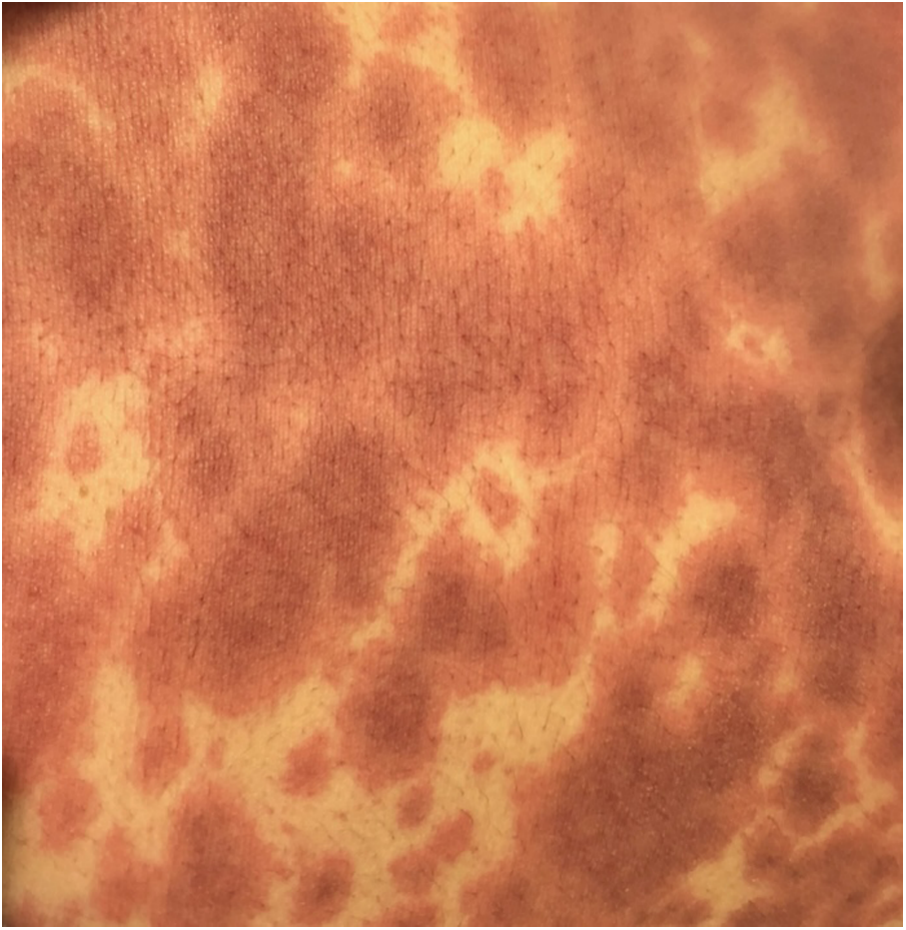
Erythema multiforme in a subject with herpes simplex virus infection.

EM has an acute presentation. It starts with a painful eruption, often an erythematous papule, that eventually progresses to form a target lesion. Its resemblance to urticaria is related to its lighter oedematous appearance surrounded by a peripheral erythematous border (Figure 2). One of the main characteristics of the target lesion is its central dusky area which is not seen in urticaria. The central area may not necessarily blister. In fact, the hallmark of the target lesion is its three distinct, concentric zones with colour change (18, 19). On the mucous membrane, the lesion may form a blister and erode, forming a crust as it heals. It may involve mucous membranes of the oral cavity, eyes, or genitals. Ocular involvement may pose serious complications, including conjunctival scarring, keratitis, and visual impairment. Recurrent cases in the paediatric cohort are commonly linked to HSV (20).

Diagnosis of EM is based on clinical examination of its characteristic morphology. It is supported by investigations that further evaluate an infective aetiology, including full blood count, inflammatory markers, polymerase chain reaction (PCR) test for HSV from a concurrent skin lesion (e.g., a cold sore), nasal PCR test for *Mycoplasma pneumoniae,* and chest x-ray looking for pulmonary infiltrates. A skin biopsy should be performed if in doubt. Characteristic histopathological findings include keratinocyte necrosis, particularly in the centre of the targetoid lesion (21). Necrosis of the whole dermis may be seen in severe cases. Direct immunofluorescence is negative.

## Urticaria vasculitis

Urticarial vasculitis (UV) is characterised by long-standing urticarial rashes and histopathologic findings of leukocytoclastic vasculitis (22). The lesion has an inflamed and brightly erythematous wheal that resembles urticaria, with the additional feature of vessel inflammation. UV being a systemic disease may involve internal organs. An array of conditions, ranging from autoimmune and infective to paraneoplastic, have been associated with UV. Autoimmune diseases [e.g., Systemic Lupus Erythematosus (SLE) and Sjögren's Syndrome], haematological diseases (e.g., lymphoproliferative diseases and monoclonal gammopathy) (23, 24), infections (e.g., Streptococcus and viral hepatitis) (25)^,^ and drugs (e.g., antibiotics) can all cause UV, though in the majority of cases, the cause is unknown.

There are two types of UV, known as normocomplementaemic UV and hypocomplementaemic UV (McDuffie syndrome). Each type has unique pathophysiology, clinical manifestations, and prognosis. UV is considered a complement-mediated disease that is considered a type-3 hypersensitivity reaction. The pathophysiology is a complex interplay between the interaction of antibodies with antigens resulting in complement cascade activation and production of C3a, C5a, and C5b-9 via the classical pathway (26, 27). Systemic involvement is more commonly seen in HUV than in NUV (28, 29).

The unique role of anti-C1q autoantibodies in hypocomplementaemic UV (HUV) is explained by the activation of complement cascade by autoantibodies targeting the collagen-like region of C1q (30). The pathogenic role of anti-C1q autoantibodies in immune complex-mediated renal disease is supported by previous studies, with further observation of level rise in anti-C1q antibodies prior to a relapse of lupus nephritis, as early as six months in advance (31, 32). Anti-C1q antibodies were found to specifically target C1q bound on cells undergoing apoptosis in a previous study involving patients with SLE, indicating that early apoptotic cells are a major target for the autoimmune response (33). Additionally, it has been postulated that pulmonary insult in HUV is a result of anti-C1q antibodies cross-reacting with pulmonary alveolar surfactant apoprotein that contains C1q collagen-like proteins, resulting in chronic obstructive airway disease (34).

The hallmark symptom of an urticarial vasculitis lesion is a painful and burning sensation, in contrast to the wheals of acute or chronic urticaria which are typically pruritic. It is a common mimic of urticaria, however, additional clues to UV include a bruised appearance, longer duration of lesions, often for days (in contrast to acute or chronic urticaria lesions that tend to resolve within a few hours), and healing with residual post-inflammatory ecchymotic hyperpigmented marks.

Constitutional symptoms, such as malaise and fever, as well as internal organ involvement, may occur. Arthralgia may be present in approximately 50% of cases (35). Any joints may be affected, commonly the joints of the upper and lower limbs, including the hands and feet. Renal involvement is not uncommon, necessitating further workup to exclude haematuria and proteinuria, and if found, further characterisation of intrinsic kidney involvement may be warranted, such as renal biopsy. Well-described associations include membranoproliferative, mesangioproliferative, and crescentic glomerulonephritis (36, 37). Pulmonary involvement may be present in UV, particularly obstructive airway diseases such as asthma and chronic obstructive airway disease. Vasculitis may also involve the gastrointestinal tract and eyes.

Clinical assessment for UV should always include a systemic review given internal organ involvement, especially in HUV. Physical examination may reveal tell-tale signs of UV, especially from the cutaneous aspect. Thorough investigations including full blood count, inflammatory markers, serum complements, renal function, and liver enzymes are considered basic. Further exploration into a potential autoimmune, infective, or neoplastic aetiology should be undertaken, including antinuclear antibodies, extractable nuclear antigens, anti-double-stranded DNA antibodies, rheumatoid factor, anti-citrullinated protein antibodies, cryoglobulins, anti-neutrophil cytoplasmic antibodies, viral hepatitis serology, anti-streptolysin O titres, full blood picture, lymphocyte immunophenotyping, serum protein electrophoresis, serum free light chain, tumour markers, and urine Bence-Jones protein. Urine analysis looking for the presence of protein, blood, or casts, should be performed. Renal biopsy may be required, especially to confirm or establish the type of glomerulonephritis. In the setting of pulmonary involvement, chest x-ray, chest computed tomography, lung function tests, and blood gas measurement are all useful. With musculoskeletal involvement, a skeletal survey is recommended.

Skin biopsy demonstrates features suggestive of leukocytoclastic vasculitis. The key feature is neutrophil predominance, in contrast to non-vasculitic urticarial samples, which may show mostly the presence of eosinophils. A novel histopathological diagnostic scoring system was established recently to distinguish UV from CSU. A combined quantitative assessment of three key features of leukocytoclasia, fibrin deposits, and extravasated erythrocytes convincingly distinguishes UV from CSU in skin histopathology reading (38). This scoring system serves as a useful tool to reduce the chance of misdiagnosis of UV.

## Erythema marginatum

Erythema marginatum (EM) is a reticular, serpiginous, erythematous lesion that may be mistaken for urticaria. Unlike urticaria, EM is not pruritic and is less widespread. EM was first described by Dinckelacker in 1882. EM is commonly associated with Hereditary Angioedema (HAE), where it may occur alone, or together with fatigue and malaise, as a prodromal symptom experienced by patients with HAE prior to an imminent acute attack of angioedema. In certain parts of the world, including Central and Northern Australia and Southeast Asia where rheumatic fever is endemic, EM is one of the major criteria for this infection caused by Streptococcus.

EM may occur on any part of the body, including the trunk and limbs. Given its morphology mimicking urticaria, it is unsurprising that patients with HAE presenting with EM and angioedema potentially may be misdiagnosed as having an allergy. Recent studies have highlighted the rate of misdiagnosis or delayed diagnosis of HAE given misinterpretation of EM as urticaria. Diagnostic delay for 2 years in HAE patients with EM had previously been found (39).

Prodromal symptoms occur hours to days before an acute attack in HAE, with almost two-thirds in a large German population study experiencing the symptoms within 6 h before an attack (40). The syndrome consists of fatigue, malaise, short temper, and EM. There was a significant association between EM and delayed diagnosis of HAE.

In the setting of HAE, EM may occur in HAE with C1 esterase inhibitor (C1-INH) deficiency as well as with normal C1-INH levels. This was observed in a Japanese survey on prodromal symptoms in patients with HAE. The same study reported EM distribution on the forearm as the predominant site, followed by the abdomen, upper arm, and precordium (41).

HAE is an inherited condition with or without C1-INH deficiency. In type 1 HAE, both C1-INH function and antigenic levels are low. In type 2 HAE, C1-INH function is low, whereas C1-INH antigenic level may be normal or elevated. Both types are caused by SERPING1 gene mutations (42). Known genetic mutations in HAE with normal C1-INH are factor XII, plasminogen, angiopoietin-1, kininogen-1, myoferlin, and heparan sulfate glucosamine 3-o-sulfotransferase 6 (43–48). Each of these mutations results in distinctive pathophysiology in HAE with normal C1INH. For example, in HAE with angiopoietin-1 the mutation alters the ability of the angiopoietin-1 gene to modulate endothelial permeability induced by vascular endothelial growth factor, resulting in vascular leakage (45).

## Urticarial dermatitis

Urticarial dermatitis (UD) is an intensely pruritic skin eruption that may exist in papules and plaques resembling urticaria. Unlike urticaria, which has a rapid resolution, UD often lasts longer than 24 h. It is recognised that UD may persist for days to even weeks (49). Common distribution includes the trunk and extremities, with the palms and soles typically spared. Like many eczematous dermatoses, chronic scratching may result in lichenification.

Skin biopsy is helpful when certain features unique to other dermatoses are absent. Overall, non-specific findings are seen on skin biopsy taken from a UD lesion. These include epidermal oedema with perivascular inflammatory (often lymphocytic) infiltrate, with eosinophils present in the papillary and upper reticular dermis (50). Dermal hypersensitivity reaction pattern is a common descriptive phrase. Importantly, prominent spongiosis when present, points towards a diagnosis of eczematous dermatoses.

## Bullous pemphigoid

Bullous pemphigoid (BP) is an autoimmune, heterogeneous, blistering skin disease affecting mostly older adults. It may involve both the skin and mucous membranes. BP is intensely pruritic in the prodromal phase, characterised by non-bullous lesion eruption, often in the form of excoriated, urticarial, or eczematous lesions (51, 52). As the disease progresses, tense blisters form on the urticarial base and become more widespread. Scarring is not usually a feature of BP as the bullae rupture and heal. Though various mucosal tissues may be involved, oral mucosa is the most frequently affected mucosal surface (53).

Skin biopsy of lesional tissue and direct immunofluorescent (DIF) staining of the perilesional tissue specimen are key investigations. Key histopathological findings include subepithelial blister formation and eosinophilic spongiosis (52). Skin biopsy interpretation of an early non-bullous lesion can be challenging because of non-specific findings (54). DIF typically demonstrates staining of linear IgG and linear C3 along the basement membrane zone in most cases. Indirect immunofluorescent (IIF) testing is utilised to detect circulating IgG antibodies along the basement membrane zone. Enzyme-linked immunosorbent assay (ELISA) detects circulating IgE antibodies against BP180 and BP230, which are pathogenic autoantigens in BP (55–57). In particular, IgE anti-BP230 antibodies have been shown to play an important role in local eosinophil accumulation in skin lesions.

## Polymorphic eruption of pregnancy

### *also known as pruritic urticarial papules and plaques of pregnancy (PUPPP)

Pruritic urticarial papules and plaques of pregnancy (PUPP) is a benign inflammatory disorder that is the most common dermatosis in pregnancy, appearing most commonly in the last weeks of pregnancy. Itchy small papules and plaques start in stretch marks and spare the periumbilical area. The eruption generally spreads to the trunk and limbs but rarely involves the face. There is no mucosal involvement. It resolves rapidly postpartum, leaving no pigmentation or scarring.

The cause of the condition is unknown but several factors are considered, including rapid abdominal distension, hormonal changes, and placental and foetal factors. One theory suggests that connective tissue damage within striae is a factor.

It is seen in primigravidas primarily and does not typically recur in later pregnancies (58). The incidence is higher in twin (3%–16%) and triplet (14%–17%) pregnancies compared to single pregnancies (0.5%). There are no familial links or underlying autoimmune diatheses identified.

Biopsy specimens from lesions show non-specific features and direct immunofluorescence is usually negative. Routine laboratory tests are within normal limits.

Symptomatic treatment is usually all that is required with topical steroids and antihistamines commonly prescribed. If severe, causing sleep disturbance, a very short course of oral corticosteroids may be considered.

Apart from the discomfort, there are no negative consequences; pregnancy and maternal prognosis are not affected (58).

Characteristic features and investigations of each of the conditions are summarised in Table 1.

**Table 1:** Summary of mimickers of urticaria.

Condition	Pathophysiology	Secondary associations	Telltale features	Investigations
Cutaneous mastocytosis	Genetic	Systemic mastocytosis	Darier's sign may be present	Skin biopsy for mast cell infiltrate Serum for c-KIT D816V analysis
Erythema multiforme	Hypersensitivity	Infections Drugs	Target lesion with a central dusky area	Inflammatory markers PCR for HSV or Mycoplasma Skin biopsy for keratinocyte necrosis
Urticaria vasculitis	Inflammatory	Autoimmune diseases Lymphoproliferative diseases Infections Drugs	Bruised lesions that heal with ecchymotic hyper-pigmented marks	Inflammatory markers Serum complements Screening for autoimmune, lymphoproliferative diseases and infections Skin biopsy for leukocytoclastic vasculitis and neutrophilic predominance
Erythema marginatum	Inflammatory	Hereditary angioedema	Often coexists with prodrome before an acute flare of angioedema	Serum C4 Serum C1 esterase inhibitor (function and antigenic levels)
Urticarial dermatitis	Inflammatory	N/A	Unlike urticaria, often lasts longer than 24 h, or even weeks	Skin biopsy for epidermal oedema, perivascular infiltrate, eosinophils
Bullous pemphigoid	Autoimmune	N/A	Tense blister forms on the urticarial base	DIF for linear IgG along BMZ IIF for IgG antibodies along with BMZ Anti-BP180 and BP230 antibodies
Polymorphic eruption of pregnancy	Inflammatory	N/A	Itchy small papules and plaques start in stretch marks and spare the periumbilical area	None is usually required given the clinical diagnosis

PCR, polymerase chain reaction; HSV, herpes simplex virus; DIF, direct immunofluorescence; IIF, indirect immunofluorescence; BMZ, basement membrane zone.

## Conclusion

There are many dermatological conditions that resemble the appearance of urticaria, making diagnosis sometimes challenging. With an understanding of underlying disease associations and pathology, together with a recognition of pathognomonic features, a diagnosis can be established with the aid of relevant investigations.
